# Rethinking rangeland and grassland management for sustaining livelihoods and ecosystem services

**DOI:** 10.3389/fpls.2026.1825430

**Published:** 2026-07-08

**Authors:** Nityananda Khanal

**Affiliations:** Agriculture and Agri-Food Canada, Beaverlodge, AB, Canada

**Keywords:** indigenous knowledge, seasonality, soil, spatial ecoengineering, species

## Abstract

Rangelands and grasslands are critical to indigenous and agriculture-based livelihoods. The sustainability of these land use systems relies on nature-friendly practices that optimise ecosystem services. This article proposes a simple “8-S” framework for the nature-friendly management of rangeland systems, including grasslands. The framework emphasises the assimilation of indigenous and modern scientific knowledge in managing space (S1), species (S2), soil and water (S3), seasonality (S4), and stress factors (S5) through a systems approach (S6). Concurrently, it emphasises the deployment of supportive infrastructure and institutional services (S7) that incentivise environmental stewardship and reinforce socioeconomic sovereignty (S8). The convergence of population pressures, evolving societal needs, finite landscape capacity, climate change, and resource overexploitation is pushing ecosystems toward tipping points, necessitating the redesign of terrestrial space through spatial ecoengineering (S1). The S1 should foster the evolution and conservation of animal, plant, and microbial species making up natural and economic biodiversity (S2); enhance soil health and hydrologic properties (S3); and enable adaptation to seasonal and climatic anomalies by moderating microclimate, preserving water resources, and adjusting management tactics (S4). Synergising the above “4-S” elements and supplementary measures imparts resilience to the system against stress factors (pests, drought, temperature extremes, wind, etc.) (S5). At the same time, a community-led co-development with a systems approach to the integration of livestock grazing, resource extraction, and recreational activities (S6), along with supportive infrastructures and policy environment, incentivises environmental stewardship (S7). These “7-S” measures help balance systems trade-offs while enhancing resource circularity. This informed and collaborative approach contributes to socioeconomic sovereignty, fostering sustainable and self-reliant livelihoods of rangeland-based communities (S8).

## Graphical Abstract

**Figure:**
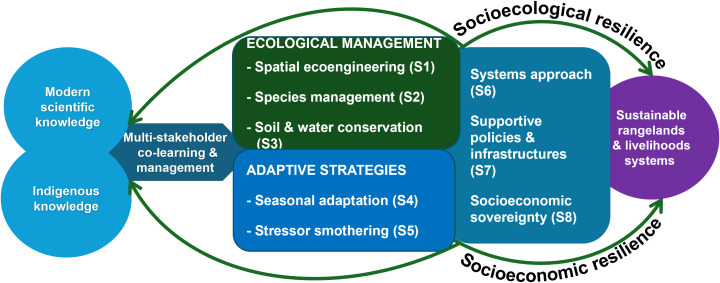
The conceptual diagram presents a sequential and adaptive representation of the 8-S framework for nature-friendly rangeland and grassland management. The ecological core (S1–S4) establishes the biophysical foundation through spatial ecoengineering, biodiversity management, soil and water conservation, and seasonal adaptation. Synergistic effects impart resilience to stressors (S5), enable integrated system functioning (S6), and are reinforced by stewardship-friendly policies and infrastructures (S7), ultimately culminating in socioeconomic rationality and sovereign livelihoods (S8). Indigenous and modern scientific knowledge, mobilised through multi-stakeholder co-learning and management practices, drives adaptive feedback that sustains socioecological and socioeconomic resilience.

## Introduction

By carrying the Anishinaabe Seven Grandfather Teachings into space aboard Artemis II, Canadian astronaut Jeremy Hanson symbolised a principle of profound contemporary relevance. Addressing today’s socioecological crises requires integrating indigenous knowledge systems with modern scientific approaches to stewardship and resilience (Olson, 2026). This message resonates strongly with the planetary boundaries framework, which conceptualises Earth as a finite life-support system with biophysical thresholds that define a safe operating space for humanity. Indigenous worldviews emphasise respect for nature’s entities and express humility through gratitude for the endowments provided to human beings and all life forms via diverse ecosystem services (Kealiikanakaoleohaililani and Giardina, 2016). These biocultural values have historically guided the management of the natural resource base (Bădărău et al., 2025; Nita et al., 2024). In contrast, the modern economic system prioritises short-term financial gains, while failing to adequately value these natural services (Kealiikanakaoleohaililani and Giardina, 2016).

Consequently, the current trajectory of global resource use and interconnected socioeconomic systems has led to a range of socioecological issues. Seven of the nine components of the planet’s life-support systems, including the climate, biosphere integrity, land use, water and nutrient cycles, atmospheric aerosols, and ocean acidification, have exceeded safe operating boundaries. Rising greenhouse gas emissions and ecosystem imbalances are accelerating global warming, pushing 16 sub-domains of the Earth system toward critical tipping points (Rockström et al., 2024, Rockström et al., 2026). As a result, the global biomes have increasingly transformed into the anthromes, reflecting widespread anthropogenic impacts (Walker et al., 2026).

The years 2023 and 2024 have set new records for global temperatures and climate-related disasters (Ripple et al., 2024). If current warming trends continue, projected losses by 2060 include a decline of 18%–37% in resources, 37%–45% in public health outcomes, and 12%–43% in labour productivity. These changes could seriously disrupt global supply chains and the way of life (Sun et al., 2024). As planetary boundaries continue to be overshot, rangeland ecosystems occupy a central role in efforts to restore planetary resilience.

The rangeland is commonly defined as the uncultivated land that provides the necessities of life for grazing and browsing animals, or as the type of land on which natural vegetation is dominated by grasses and shrubs and the land is managed as a natural ecosystem (Briske, 2017). The functional services of rangeland ecosystems, which also include grasslands, encompass a wide range of ecological and socioeconomic benefits. They serve as sources of food, medicines, livestock feed, and diverse non-timber products, thereby forming an important basis of livelihoods for millions of people in the world (Godde et al., 2020). Moreover, rangelands occupy 54% of the terrestrial surface area (Onyango et al., 2022) and function as vast carbon sinks, playing a significant role in global carbon sequestration (Schuman et al., 2002). However, excessive extraction of resources and associated industrial activities have caused the degradation of these lands’ resources. This is reinforcing climate change and environmental pollution, while accelerating biogeochemical cycles. These impacts have led to the loss of native biodiversity and the spread of invasive species, posing threats to indigenous livelihoods (Roberts et al., 2024).

The dynamics of rangeland and grassland systems are inherently complex. Over the century, the rangelands have undergone increasingly intense anthropogenic impacts across geophysical, ecological, and socioeconomic dimensions. In response, rangeland management paradigms have evolved from equilibrium-based models of vegetation succession and non-equilibrium state-and-transition frameworks toward ecosystem resilience-based approaches. These contemporary approaches focus on sustaining livestock production under conditions of variability and change. However, the dominant livestock productivism narrows the conceptualisation of rangelands by sidelining a wide range of ecosystem services within dynamic socioecological systems (Bestelmeyer and Briske, 2012; Ghorbani et al., 2013). Moreover, the top-down administrative management paradigms largely shaped by academic ecological theories have inadvertently undermined the indigenous knowledge systems that have guided rangeland stewardship for millennia (Ghorbani et al., 2013). As a result, the priorities and interests of local communities have been marginalised (Mantyka-Pringle et al., 2025).

This perspective article proposes a simple operational framework for nature-friendly and community-empowering rangeland and grassland management systems Khanal (2023). The novelty of the proposed 8-S framework lies in its holistic integration of ecological, spatial, cultural, institutional, and socioeconomic dimensions into a single systems-based approach. Unlike the predominant technocentric, top-down rangeland and grassland management frameworks (Bennett et al., 2023; Briske, 2017), it introduces a simplified concept of spatial ecoengineering, explicitly fuses indigenous and scientific knowledge, and incorporates supportive infrastructure and socioeconomic sovereignty as core components. The framework’s sequential and systemic structure (S1–S8) provides a comprehensive, actionable, stakeholder-comprehensible, and participatory approach to rangeland management by linking ecological resilience with community-led stewardship and sustainable livelihoods.

## Methods

Positioned at the interface of practice and scholarship, and informed by lived experience across diverse farming communities where rangelands were integral to agricultural systems, the author brings a practitioner-informed and justice-oriented perspective to this study. This study adopts a conceptual synthesis that builds upon and extends experiential insights originally proposed by Khanal (2023). It draws on a purposefully curated, transdisciplinary body of literature to examine the integration of indigenous agrarian knowledge with modern scientific inquiry in rangeland management.

Rather than being driven primarily by systematic database queries, the synthesis selectively engages with relevant literature identified through Google Scholar, focussing on publications between 2000 and 2026. Key search terms included climate change, planetary boundaries, grassland systems, rangeland management, and rangeland-based livelihoods, alongside spatial ecoengineering, biodiversity, soil and water resource conservation, seasonal adaptation, stressor management, integrated pest management, integration of indigenous knowledge and modern scientific knowledge, and socioecological justice. A comprehensive literature review identified 69 relevant publications that substantiate and illustrate the author-preconceived 8-S framework, emphasising practitioner-relevant insights, contemporary discourse, and policy-relevant terminology. Those publications are fully referenced and compiled in **Table 1**, organised into 11 thematic areas.

**Table 1:** Literature underpinning the conceptual synthesis, categorised by thematic areas.

Thematic areas	Cited literature
Indigenous knowledge system; knowledge integration	Olson (2026), Kealiikanakaoleohaililani and Giardina (2016), Bădărău et al. (2025), Nita et al. (2024), Ghorbani et al. (2013), Mantyka-Pringle et al. (2025), Bohensky and Maru (2011), Hill et al. (2020), Hird et al. (2023)
Planetary boundaries, Earth system justice, and climate change impacts	Rockström et al. (2024), Rockström et al. (2026), Gupta et al. (2024), Walker et al. (2026), Ripple et al. (2024), Sun et al. (2024)
Rangeland ecosystems; management models, approaches, and impacts	Briske (2017), Godde et al. (2020), Onyango et al. (2022), Schuman et al. (2002), Roberts et al. (2024), Bestelmeyer and Briske (2012), Bennett et al. (2023), Reeves et al. (2018), Reid et al. (2014)
Spatial ecoengineering	Scherr et al. (2012), Dollinger et al. (2015), Gobinath et al. (2022), Rey et al. (2019), Gayathiri et al. (2022), Ray et al. (2023), Teixeira et al. (2023), Schmitt et al. (2018), Booze-Daniels et al. (2000), Gobinath et al. (2024), Niborski et al. (2023), Gurrieri (2020), Wu et al. (2025), Khanal (2023) [cited above: Reeves et al. (2018), Reid et al. (2014)]
Species management	Soder et al. (2007), Alders et al. (2021), Derebe et al. (2023), Gautam et al. (2022), Asbjornsen et al. (2014), Bestelmeyer et al. (2024), McCord and Pilliod (2022), Sherrill et al. (2022)
Soil and water conservation	Dyer et al. (2021), Kassahun et al. (2008) [cited above: Bestelmeyer et al. (2024), McCord and Pilliod (2022)]
Seasonal adaptation	Trail and Ward (2024), Vermeire et al. (2023), Bhusal and Awasthi (2024), Aryal et al. (2014) [cited above: Bestelmeyer et al. (2024), McCord and Pilliod (2022), Bohensky and Maru (2011)]
Stressor buffering	Luxom et al. (2022), Li et al. (2023), Cumming and Spiesman (2006), Krueger-Mangold et al. (2006)
Systems approach	Briske et al. (2020), Plieninger and Huntsinger (2018), Fox et al. (2009), Petursdottir et al. (2013), Niamir-Fuller and Huber-Sannwald (2020)
Stewardship-friendly policies, support services, and infrastructures	Puntenney et al. (2025), Falayi et al. (2022), Robinson et al. (2017), Robinson et al. (2021) [cited above: Petursdottir et al. (2013), Briske et al. (2020)]
Socioeconomic sovereignty	Mendes dos Santos et al. (2026), Van Assche et al. (2017), Vallet et al. (2020) [cited above: Hill et al (2020), Hird et al. (2023), Reid et al. (2014)]

While grounded in and supported by the existing body of literature, the proposed framework does not constitute a formal systematic literature review. This is acknowledged as a limitation of the study.

## Results and discussion

Whether it is cropping systems, or grassland or rangeland systems, their sustainability hinges on the integration of sound ecological and Earth system justice principles, encompassing inter-societal, inter-generational, and inter-species justice (Gupta et al., 2024). Aligning with those principles, the proposed framework identifies eight operational elements for nature-friendly rangeland and grassland management and sustainable livelihood systems. Central to the framework is the assimilation of indigenous and modern scientific knowledge in managing key ecological dimensions: space (S1), species (S2), soil and water (S3), seasonality (S4), and stress factors (S5). These five ecological components are operationalised through a participatory systems approach (S6) and are supported by enabling infrastructure and institutional services (S7) that incentivise environmental stewardship while reinforcing socioeconomic sovereignty (S8). Collectively, these eight interconnected operational elements constitute the “8-S” framework.

The framework recognises spatial ecoengineering (S1) as a foundational configuration for fostering the evolution and conservation of animal, plant, and microbial species making up natural and economic biodiversity (S2); enhancing soil health and hydrologic properties (S3); and enabling adaptation to seasonal anomalies and climate change by moderating microclimate, preserving water resources, and adjusting management tactics (S4). Synergising the above “4-S” elements and supplementary measures imparts resilience to the system against stress factors (pests, drought, temperature extremes, wind, etc.) (S5). At the same time, a community-led co-development with a systems approach to the integration of livestock grazing, resource-extractive, and recreational activities (S6), along with a supportive policy environment, incentivises environmental stewardship (S7). This, in turn, balances systems trade-offs and improves systems synergy by circularising the flow of resources. This informed and collaborative approach contributes to socioeconomic sovereignty with sustainable livelihoods of rangeland- and grassland-based communities (S8). This systems-based framework integrates socioecological dynamics by considering social (community, policy, and institutions) and natural (soil, water, plants, and animals) components as interacting and interdependent elements. It emphasises collaborative governance, community-led co-development, and resource flow circularity to enhance system feedback, thereby improving adaptation and long-term ecosystem viability and socioecological synergy, rather than maximising commodity outputs (such as meat, milk, and wool production). The discussion elaborates on the 8-S elements supported by contemporary literature and examples drawn from indigenous rangeland management contexts.

### S1: spatial ecoengineering

Ecological heterogeneity is a ground reality of all rangelands and grasslands. Growing human population, developmental pressures, and changing land use and land tenures have led to increased diversification and intensification, land fragmentation, landscape modification, degradation, and desertification (Reeves et al., 2018; Reid et al., 2014). The construction of developmental infrastructures, including transportation corridors and industrial resource extractions, has altered drainage systems and landscape configuration. Moreover, ecosystems have been vulnerable to inclement weather patterns as manifestations of climate change. In these anthropogenic and climatic pressures, merely managing the grazing and resource harvest schedules is not sufficient. The holistic management framework must embrace adaptive and dynamic configuration of landscape and vegetational features, which is here termed spatial ecoengineering. Other terminologies used in the literature to refer to similar management features include climate-smart landscapes (Scherr et al., 2012), agroecological engineering (Dollinger et al., 2015), ecoengineering (Gobinath et al., 2022), and soil and water bioengineering (Rey et al., 2019). The spatial ecoengineering involves the application of landscape stabilisation, rehabilitation, augmentation, and adaptive conservation measures tailored to the topographical and vegetational attributes to enhance microclimate, improve ecosystem health, conserve biodiversity, and regulate local hydrological systems (Gayathiri et al., 2022; Ray et al., 2023).

Indigenous communities historically employed diverse landscape and vegetational augmentation measures in and around the rangelands to enhance productive, protective, cultural, and recreational needs (Appendix 1). These included strategic nurturing or planting of trees, establishment of water-harvesting ponds, and building dikes or retaining walls. The trees served specialised or multipurpose roles such as producing fruits, providing shade to grazing animals during hot days, stabilising slopes or stream banks, and/or producing supplemental fodder or bedding materials for livestock. The ponds and reservoirs would not only provide drinking water for animals but also help recharge downstream water springs. The dikes and retaining walls with tailored vegetation controlled erosion, stabilised vulnerable landscapes, and served as the delineation of land use systems and areas of beneficial biodiversity habitats. Collectively, these traditional ecoengineering features moderated microclimate, augmenting land productivity and overall ecosystem services. Ecological embankment with restored river dike grasslands in Germany is a special example of a traditional ecoengineering feature (Teixeira et al., 2023). Contemporarily, digitised land use zoning based on geomorphic capabilities can enhance participatory spatial ecoengineering capabilities. Such zoning can guide niche-based adaptation of economic activities, such as the frequency and intensity of grazing and harvest of non-timber forest products. It also helps identify leverage points for ecological conservation and resource augmentation measures. These include land-stabilising native plantations along river banks (Schmitt et al., 2018), transportation and drainage corridors (Booze-Daniels et al., 2000), and areas prone to landslides and soil erosion (Gobinath et al., 2024), as well as ecological embankment (Teixeira et al., 2023), rainwater harvesting (Niborski et al., 2023), water development at springs (Gurrieri, 2020), and the delineation of areas for protecting beneficial biodiversity habitats (Wu et al., 2025). Various examples of traditional and contemporary spatial ecoengineering practices around the world illustrate key management elements applicable to sustainable rangeland management.

### S2: species management

Biodiversity forms a fundamental basis for sustaining rangeland and grassland ecosystems. Plant diversity, occupying diverse ecological niches, serves as a primary source of nutrition for livestock and meets the varied preferences and nutritional needs of both browsing and grazing animals (Soder et al., 2007). Moreover, both plant diversity and animal diversity provide essential resources, including food, medicines, other non-timber products, and diversified income opportunities for local communities (Alders et al., 2021; Derebe et al., 2023). According to Gautam et al. (2022), communities in Nepal harvest edible plant products from 436 species, including 208 herbs, 68 shrubs, 120 trees, and 40 climbers. Diverse perennial vegetation contributes to a wide range of ecosystem services, including soil and water conservation, hydrological regulation, nutrient cycling, carbon sequestration, air purification, aesthetic value, microclimate regulation, and resilience to extreme disturbances, thereby sustaining overall ecosystem functions (Asbjornsen et al., 2014). Thus, biodiversity is intrinsically linked to both human livelihoods and the functioning of entire ecosystems.

Loss of biodiversity is a burgeoning concern across all global biomes in the world. Therefore, species management should become one of the primary goals of rangeland and grassland management. Within the locally tailored spatial ecoengineering framework, interventions such as regenerative grazing, strategic plant regeneration and planting, and restoration interventions, co-developed through multi-stakeholder participation, can play a pivotal role in maintaining and enhancing species diversity in the rangelands. Furthermore, the development of systematic inventories, long-term monitoring systems, and analytical databases of all native fauna and biota is essential. Such efforts lead to success with the active stake of the local community and the integration of indigenous knowledge (Bestelmeyer et al., 2024; McCord and Pilliod, 2022). At the same time, concerted vigilance and efforts should be accorded to exclude and eradicate invasive species (Sherrill et al., 2022). Special conservation strategies should be in place for the regeneration of endangered and declining species and for the reintroduction of extirpated species with the community-based regulation of grazing and harvesting activities.

### S3: soil and water conservation

The maintenance of soil, water, and ecosystem health is critical for sustaining rangeland and grassland productivity (Dyer et al., 2021). Mismanagement of rangelands, such as inappropriate grazing intensity and timing, and a lack of a proactive approach to protect fragile areas, can trigger the cascading degradation of soil and water resources. Such degradation manifests in soil erosion, gully formation, landslides, scouring of streambanks and drainage corridors, reduced recharge of natural springs, increased sediment loads in rivers, and downstream flooding. These impacts lead to a substantial decline in rangeland productivity and associated ecosystem services, while increasing system vulnerability to climate change. Consequently, this threatens rangeland-dependent livelihoods (Kassahun et al., 2008). Improving soil properties and regulating hydrological and biogeochemical cycles are fundamental to the sustainability of rangeland socioecological systems. Therefore, systematic monitoring and studies on soil health, as well as hydrological and biogeochemical indicators, are essential to understand the impacts of prevalent practices, inform adaptive resource use strategies, and guide the implementation of regenerative solutions (Bestelmeyer et al., 2024; McCord and Pilliod, 2022). An extensive body of literature documents and substantiates the need, strategies, and impacts of soil and water conservation interventions.

### S4: seasonal adaptation

It involves tailoring grazing and resource-extractive practices to seasonal rhythms in accordance with spatial microclimatic features and prevailing weather patterns (Trail and Ward, 2024; Vermeire et al., 2023). Traditional pastoralists in the high-altitude regions of Nepal exemplify this approach through transhumant livestock movement structured around the *Ubhauli* (ascending) and *Udhauli* (descending) seasonal cycles. These cycles align grazing intensity and timing with vegetation phenology and climatic gradients.

As temperatures rise and day length increases in spring, herds are gradually moved to higher elevations (hence *Ubhauli*), typically beginning on a full-moon day in late April (spring season). Grazing progressively extends to the highest-elevation meadows through July–August (summer). The descent (*Udhauli*) begins on full-moon day in late August (autumn), with herds gradually returning to lower elevations and reaching permanent settlements by November–December. Transhumance typically pauses during winter from December to early April.

For balancing grazing pressure, different herder groups follow distinct pre-negotiated transhumance routes and grazing destinations, which are rotated over the years. However, climate change, manifested through increased weather vagaries, has recently disrupted the predictability of these finely tuned seasonal practices (Bhusal and Awasthi, 2024). Integrating weather forecasting and agro-climatic modelling solutions (Bestelmeyer et al., 2024; McCord and Pilliod, 2022) with indigenous knowledge systems (Bohensky and Maru, 2011) can enhance adaptive, seasonally responsive decision-making. Additionally, adjusting the calendar of complementary economic activities, market engagement, and mobility patterns can help safeguard lives, livelihoods, and resource bases (Aryal et al., 2014).

### S5: stressor buffering

Stressor buffering addresses the mitigation of both biotic and abiotic stresses that can disrupt rangeland livelihood systems (Luxom et al., 2022). Climate change has intensified the frequency and severity of abiotic stresses, such as extreme temperatures and drought, which destabilise biodiversity and ecological processes. The integration of the four preceding operational elements (S1–S4) can help alleviate abiotic stress and enhance system resilience. Complementary interventions, including rangeland-appropriate insurance policies, livestock vaccination, and access to veterinary services, further strengthen the capacity to buffer and adapt to impacts of stressors.

It is also paramount to regularly monitor biotic stressors, including pests, parasites, pathogens, weeds, and invasive species affecting grazing herds, beneficial plant communities, and other rangeland biodiversity. Systematic surveillance enables timely and proactive actions to mitigate their disruptive impacts on livestock and rangeland biodiversity (Li et al., 2023). In addition, studies should focus on understanding the dynamics of these stressors and enhancing their predictability to inform targeted interventions with appropriate timing, stage, frequency, methods, and management strategies.

Integrated pest management (IPM) strategies should be developed based on historical trends and predictive assessments of the prevalence of insect pests, diseases, weeds, and invasive species. These strategies may be broadly categorised into physical (e.g., mechanical control, solarisation, anaerobic soil disinfection, controlled burning, flaming, steaming, electrocution, and the application of electrostatic fields, lasers, infrared, and ultraviolet radiation), biological (e.g., bio-fumigation and the use of botanicals), cultural (e.g., adjustment to grazing regimes), and chemical (e.g., pesticide application) approaches, selected based on feasibility and effectiveness (Cumming and Spiesman, 2006; Krueger-Mangold et al., 2006). In order to fend off predators, contemporary measures include solar or bio-fencing, the use of guard animals (e.g., farm dogs), AI-security alarms, and community-supported patrolling against problematic wildlife. Physical, biological, and cultural approaches are generally preferred over chemical measures to minimise unintended spillover effects on the environment and beneficial biodiversity. However, effective implementation requires that these strategies be co-developed by interdisciplinary experts and end-user stakeholders, taking into account local feasibility, cost-effectiveness, and scalability.

### S6: systems approach

Systems approach to community-led co-learning and rangeland management emphasises the integration of livestock grazing, resource extraction, and recreational activities (Briske et al., 2020). Indigenous knowledge and traditional wisdom provide critical leverage points to tailor modern scientific knowledge to the local ground realities. The integration of these complementary knowledge systems facilitates a comprehensive understanding of the system drivers and interactions, enabling the management of trade-offs and circularising the flow of resources (Plieninger and Huntsinger, 2018).

A participatory systems approach is to integrate ecological (S1 to S5), sociopolitical (S7), and socioeconomic (S8) disciplines (Fox et al., 2009; Petursdottir et al., 2013) in close alignment with the perspectives of rangeland users. This integration transforms the conventional rangeland management paradigm, shifting the focus away from yield maximisation and short-term economic gains toward the optimisation of ecosystem services and the enhancement of system resilience (Niamir-Fuller and Huber-Sannwald, 2020). Such an approach resonates with the foundational principles of sustainable livelihoods and fosters adaptive capacity, along with a sense of stewardship, responsibility, and reciprocity toward nature and its essential services to both people and other living beings.

### S7: stewardship-friendly policies, support services, and infrastructures

The challenges confronting rangeland and grassland ecosystems and the solutions required to address and adapt to them vary across sites depending on biophysical and socioeconomic circumstances. Accordingly, ranchers whose perspectives are shaped by local experience and place-based knowledge often prioritise management objectives that differ from those of governmental rangeland managers. Such divergence has been observed in the contrasting mental models of rangeland management across environmental gradients in the United States (Puntenney et al., 2025). Consequently, externally driven, top-down policies and management approaches have frequently proven ineffective and, in some cases, have exacerbated rangeland degradation and destabilised resource-based livelihood systems (Falayi et al., 2022; Petursdottir et al., 2013; Robinson et al., 2017). The realisation of these limitations has led to greater emphasis on multi-stakeholder participation, bottom-up approaches to policy-making, and the development of community-based conservation initiatives (Boyd and Svejcar, 2009).

A supportive policy environment is critical for incentivising environmental stewardship. Due recognition and the integration of indigenous knowledge augment participatory processes and empower primary rangeland stakeholders (Robinson et al., 2021). Furthermore, the creation of enabling environments, supported by appropriate infrastructures, institutional frameworks, and service delivery systems, is fundamental to fostering resilient and self-reliant rangeland- and grassland-based livelihood systems (Briske et al., 2020). Policy-making and program development should therefore be grounded in bottom-up, participatory approaches, rather than relying on top-down, trickle-down impositions.

### S8: socioeconomic sovereignty

Historically, pastoralist communities have been attempting to align their rangeland-based activities with their livelihood objectives and wellbeing, including income, social obligations, leisure, and recreation (Reid et al., 2014). However, persistent power asymmetry among stakeholders, along with the marginalisation of time-tested indigenous knowledge systems in policy-making, research, and development processes, continues to constrain the fair stake and equitable participation of the rangeland- and grassland-based communities (Mendes dos Santos et al., 2026; Van Assche et al., 2017). This imbalance perpetuates cycles of degradation and vulnerability. Consequently, mechanisms that assimilate indigenous and modern scientific knowledge (Hill et al., 2020), alongside deliberate efforts to address power and knowledge imbalances, are essential for sustaining rangeland- and grassland-reliant livelihoods and ecosystem services (Hird et al., 2023).

A sagacity in and support to sovereign rangeland-based livelihood systems entail the recognition and abolishment of policy bias and power asymmetry (Vallet et al., 2020). Strengthening sovereign, community-led livelihood systems necessitates the operationalisation of bottom-up scaling of policies from the community level to the national level. The policies should incentivise the rangeland communities with targeted support measures, including subsidies and compensation schemes, to promote practices that generate public goods and ecosystem services.

Overall, the study presents a novel framework integrating stakeholders’ indigenous knowledge with modern scientific knowledge for nature-friendly rangeland and grassland management, supporting sustainable rural livelihoods. The framework is grounded in the author’s cumulative experiential understanding and informed by relevant literature. While it is supported by the existing body of literature, it is a conceptual synthesis and therefore does not constitute a formal systematic literature review, which is acknowledged as a limitation of the study.

## Conclusion

The nuanced understanding of the socioecological complexity of rangeland and grassland systems is a foundational prerequisite for designing sustainable, user-empowering policies and adaptive practices. This necessitates a paradigm shift away from conventional, predominantly academic and top-down policy frameworks toward approaches that recognise the complementarity between indigenous and modern scientific knowledge systems. Such a shift emphasises participatory learning, co-production of knowledge, and community-led, collaborative development of locally adaptive, sustainable, and livelihood-supportive policies and practices.

By integrating experiential insights with accumulated transdisciplinary perspectives, this study consolidates previously fragmented knowledge into a unified 8-S operational framework for rangeland management. Advancing beyond prior frameworks, it explicitly incorporates spatial ecoengineering and stressor buffering as transdisciplinary operational components. These elements not only complement other core components, such as biodiversity and soil and water conservation, but also function as critical nodal linkages for conserving and augmenting landscape structural and ecological attributes.

Through the assimilation of the complementary knowledge systems, the proposed framework offers a simplified yet integrative structure for identifying context-specific, incremental interventions grounded in a socioecological systems perspective. By reinforcing sovereign, rangeland-reliant livelihood systems, the 8-S framework contributes to both socioecological and socioeconomic resilience. As such, the framework advances ongoing scholarly discourse while providing a practical conceptual basis for informing policy development, guiding the design of locally tailored rangeland and grassland management strategies, and supporting the implementation of site-specific programs.

## Data Availability

The original contributions presented in the study are included in the article/supplementary material, further inquiries can be directed to the corresponding author/s.

## Appendix 1: Ingenuity of indigenous wisdom seen through 8-S rangeland management framework.

A case from Thuma range in Kerunga, Arghakhanchi, Nepal is presented to illustrate the holistic nature of indigenous rangeland management practices in hilly landscapes. Management practices reflect spiritual and morally-grounded, value-based experiential wisdom passed down over generations, and often rooted to ancient *vedic* teachings.

**Spatial ecoengineering**: Traditional communal grassland systems in Nepal’s hill region were spatially configured to enhance ecological function and social cohesion. Animal drinking ponds were strategically established along ridges and near water springs, where they served multiple functions, including moderating runoff, harvesting rain water, and recharging downstream springs. Alongside these water-harvesting structures, raised resting platforms made of stones and soil, locally known as *Chautari*, were built under the shade of strategically planted sacred trees, including *Ficus religiosa*, *F. benjamina*, and *F. bengalensis.* Similarly, areas surrounding water spring were carefully managed through the protection and nurturing of naturally regenerated and/or deliberately planted vegetation.

These features provided microclimatic shades for livestock while also serving as social nodes where herders could rest, exchange knowledge, engage in recreational activities, convene meetings to reinforce collective stewardship norms, and resolve disputes. Gullies, stream corridors and riparian areas were stabilized using plant species known for their effectiveness in mitigating soil erosion and water scouring actions (e.g., *Salix* spp., *Bambusa* spp., *Vitex negundo*). Communal mango orchards and clusters of fodder tree plantations were also common features in these rangelands.

Private grasslands adjacent to communal rangelands were protected using live-fences composed of multipurpose plant species such as *Justicia adhatoda*, *Falconeria insignis*, and *Jatropha curcas*, often reinforced with thorny plant inserts and bamboo liners. These systems supported cut-and-carry practices for green forage and hay production. During rainfall events, nutrient-enriched, gentle runoff and subsurface seepage from rangeland slopes were fed into small irrigation canals supplying privately owned, diked rice fields in downstream valleys. Overall, this traditional spatial ecoengineering design thus ensured the integration of diverse elements, thereby enhancing nutrient recycling, ecological niche diversification, resource conservation and maintenance of biocultural practices.

**Species management**: Rangelands were predominantly composed of natural meadows interspersed with trees at various densities. Cultural teaching instilled a deep sense of gratitude toward nature, whereby actions such as cutting trees, disturbing soil, and tempering with water springs were accompanied by expressions of respect and forgiveness. Species diversification through community-driven or philanthropic planting of sacred, fruit-bearing, fodder, and multipurpose trees along gullies and runoff corridors was strongly encouraged. This ethos is reflected in traditional proverbs such as “having a tree is like having ten sons”, and “sowing 100 species (*sata beej*) a year is a sacred act”.

Managed vegetation and strategic plantations served multiple functions, including the provision of supplemental food (fruits, nuts, vegetables, and yams), medicinal resources, livestock fodder, shade during hot days, and regulation of microclimate, and delineation of landscape boundaries. Planting of fruit trees such as mangoes and creating resting platform beneath strategically grown sacred trees (*Ficus religiosa*, *F. benjamina*, *F. bengalensis*) were regarded as egalitarian acts of spiritual merits. Furthermore, maintaining mixed herds of cattle, buffalos, sheep and goats not only diversified household income, but also optimized the use of complementary grazing and browsing niches, thereby distributing grazing pressure more evenly across the landscape.

**Soil and water conservation**: Soil and water conservations were achieved through the integration of spatial ecoengineering design as stated above, coupled with species selection and seasonally attuned management practices. Niche-based establishment of vegetation such as perennial grass (e.g., *Eulaliopsis binate, Thysanolaena maxima*), bamboos (*Bambusa* spp.) and nurturance or plantations of diverse tree species at vulnerable landscapes helped stabilize slopes, enhance water infiltration, and facilitate nutrients cycling. Live fences composed of long-lived perennials and thorny shrubs further reinforced soil retention and landscape stability. Additional measures, such as rainwater harvesting, runoff diversion, targeted plantations around water springs along with land stabilization and erosion control strategies, contributed to water recharge and conservation. Collectively these practices supported the long-term sustainability of soil and water resources within the rangeland system.

**Seasonality**: Following the harvest of crops and herbaceous forages, livestock were also allowed to free-range on seasonal fallows and private grasslands, contributing to weed suppression, nutrient cycling, and bush control. Fodder trees planted along rangeland contours, slope edges, fences, drainage corridors and streambanks provided supplementary feed during dry periods. These resources not only supported livestock nutrition but also alleviated grazing pressure on natural meadows. Although livestock rearing was largely sedentary, animals were periodically moved to different locations to optimize the use of available forage and fodder resources, while facilitating the distribution of manure to adjacent cultivated field. Seasonal mobility and decisions were guided by traditional knowledge systems, including astronomy, almanacs and lunar calendars. Mobility were often synchronized with full-moon phases within the fortnightly cycles, enabling improved visibility, enhanced safety, and reduced risk during night movements and transitions. These practices functioned both as early predictive tools and as pragmatic adaptations to local environmental conditions.

**Stressor buffering:** Predator pressure and other disturbances were addressed through low-cost, locally adapted strategies. Livestock keepers relied on locally available herbal remedies to heal animals from illness and injuries. Predation risks were mitigated through non-lethal measures such as blowing conch shells and bells, beating metal containers to produce sharp noises, deploying guard dogs, fitting livestock with bells to enhance group cohesion and early predator detection, and construction of ambush pits in vulnerable zones. These measures exemplify culturally embedded, non-lethal stress mitigation strategies that predate formal integrated pest management or wildlife management approaches, reflecting a nuance understanding of coexistence with ecological stressors.

**Systems approach:** Crop farming, livestock keeping and rangeland management were integral component of agricultural systems that sustained rural livelihoods. The system functioned as a tightly coupled circular economy, ensuring efficient flow of nutrients across its components. Livestock played a central integrative role by converting the crop residues, weeds, and perennial rangeland vegetation into valuable outputs, while returning nutrients to the system through urine and manure.

**Stewardship and socioeconomic sovereignty**: Communities practised seasonally-attuned livestock relocation and fodder utilization strategies to prevent overgrazing and ensure sustainable resource use. Access to grazing areas and collection of non-timber products were regulated by locally established, informal community norms. Collective decision-making guided coordinated efforts in land, vegetation, soil and water management, as well as in protecting livestock from predators. Importantly, socially responsive mechanisms were embedded within these systems, whereby households facing special needs were granted conditional access to additional resources or exemptions from communal obligations through consensus-based decisions.

**Developmental paradox**: This exemplary rangeland system has largely transitioned into a historical narrative due to a confluence of factors, including inappropriate government policies promoting extensive pine plantations, the conversion of rangeland areas for infrastructural development such as vocational training institute, and the outmigration of youth for employment. The expansion of forest crop through pine plantation in the communal rangelands reduced pasture and fodder resources. This mismatched plantation, coupled with the abandonment of private crop lands due to youth migration, has led to increased forest encroachment and a rise of human-wildlife interactions, particularly involving predators such as leopards. This transformation underscores a developmental paradox, where interventions designed through top-down policy framework have inadvertently disrupted traditional socio-ecological systems. Nevertheless, these landscapes continue to offer critical design insights for reenvisioning contemporary regenerative rangeland systems.
